# Risk Management Assessments and Recommendations Among Students, Staffs, and Health Care Workers in Educational Biomedical Laboratories

**DOI:** 10.2147/RMHP.S278162

**Published:** 2021-01-15

**Authors:** Wasaif AlShammari, Hashim Alhussain, Nasser M Rizk

**Affiliations:** 1 Biomedical Sciences Department, College of Health Sciences, QU-Health, Qatar University, Doha, Qatar; 2 Biomedical Research Center (BRC), Qatar University, Doha, Qatar; 3 Biomedical and Pharmaceutical Research Unit, QU- Health, Qatar University, Doha, Qatar

**Keywords:** risk management, education laboratories, failure modes and effects analysis, risk priority number, risk control

## Abstract

**Background:**

Safety in laboratories is one of the most crucial topics for all educational institutes. All-hazards need to be identified, evaluated, and controlled whenever possible, following the risk management (RM) process. This study evaluates two academic laboratories’ risks and safety in the Department of Biomedical Science (BMS) at Qatar University (QU). The goal is to eliminate or reduce any risks to the students, teaching assistants, laboratory technicians, faculties, and other related workers, following an RM process.

**Methods:**

A cross-sectional study was performed from January to March 2020 in the BMS at QU. The study sample comprised of microbiology and hematology laboratories. Checklists and data collection sheets were used for data collection. Hazard evaluation failure mode and effects analysis (FMEA) was used. The risk priority number (RPN) was calculated for all the identified hazards. For hazard control, the hierarchy of controls was followed.

**Results:**

The number of identified hazards was thirteen (n=13) in the hematology laboratory and sixteen (n=16) in the microbiology laboratory. Chemical and ergonomic hazards had the highest percentages in both laboratories, with 25% in the microbiology laboratory and 31% in the hematology laboratory. Both laboratories were free from radiation hazards. There is a significant difference between adopted and recommended control measures in each laboratory in terms of likelihood, severity, and risk priority number (RPN).

**Conclusion:**

Both chemical and ergonomic hazards account for almost a quarter of the hazards in both laboratories. The recommended control measure can decrease the severity and likelihood of identified hazards.

## Background

Laboratories are exposed to diverse types of hazards, such as biological, chemical, and radioactive, making the laboratory a high-risk environment. The laboratory’s working environment could be exposed to several types of hazards simultaneously, which increases the risks.^1^

Scientific institutions such as universities are responsible for ensuring the safety of the environment, campus, students, faculties, staff, and laboratory workers. All personnel working inside the laboratory should be fully aware of the risks and hazards imposed by the materials and devices used. Many substances are toxic, carcinogenic, or irritating to the biological membranes, and other materials are flammable or carry the risk of biological contaminations such as samples, microorganisms, and genetic materials, in addition to other forms of risks.^2^ Proper furniture, laboratory design, and the use of appropriate devices are essential to facilitate the laboratory work and decrease risks such as ergonomic and mechanical hazards.

Safety is everyone’s responsibility. Therefore, all laboratory personnel must adhere to safety instructions to protect themselves, work colleges, and the external environment. Until the last two decades, laboratory workers and users were exposed to grave risks resulting from working without following safety procedures.^3^ Moreover, the chance of the spread of hazards outside the laboratory was a concealed problem because medical waste was not appropriately disposed of.^4^

Risk management is a process of evaluating risks and developing strategies for managing them.^5^ The risk management process comprises five steps, which are preparation, risk identification, risk assessment, risk control, and record-keeping and review.^6^ Identification of risks is the most critical step in the whole process. The risk can be identified using multiple techniques such as system mapping approaches (SMAs), structured brainstorming,^7^ previous incident reports, and consulting experts. After identifying the potential risks, the evaluation process should be conducted using various techniques such as “Failure Mode and Effects Analysis (FMEA).” FMEA was developed and adopted from the outside the healthcare, specifically the industrial sector. However, it gains more attention recently because it is beneficial to the health care system and providers as well as patients. FMEA predicts the severity and likelihood of hazards before accidents occur inside the laboratory.^8^ Attempts then should be made to control the risk following a hierarchy, which “includes elimination, substitution, engineering control, administrative controls, and personal protective equipment (PPE)”.^9^ Keeping records of the risk management process is similarly essential to show the fulfillment of safety regulations.^10^

There are three main advantages of FMEA, which track the hazards (likelihood and severity), so it can provide valuable knowledge for the future. Besides, FMEA is an indication of the predominant hazards that should receive considerable attention. Also, using FMEA actions can be taken to reduce or eliminate hazards depending on the RPN.^11^

The limitations of FMEA are subjective, tedious, and time-consuming analysis, so it requires good teamwork. A limited number of individuals knows FMEA at the expert level, but it is unknown at the enterprise level, so an experienced person should be involved in any FMEA process to add his perspective. Also, in a situation where FMEA is used, the relationship between different hazards is disregarded. Usually, FMEA is applied too late; thus, it does not affect the decision-making process. Some hazards may be missed as it depends on the experience of the individuals. The customizing and generic rating scales make the rating scale not meaning full and confusing for some individuals. The students and regular staff are often not involved in the FMEA; however, they may have a better view of hazards than internal personnel.^12^

Education laboratories play a significant role in the learning process because they offer practical experience and excellent training to students. Students who engage in well-designed laboratory experiences develop problem-solving skills and critical thinking. In addition, students exposure to various materials, and equipment in a laboratory, enhance their knowledge and awareness about risks and safety in laboratories (West, 2007). The importance of having suitable and well-established safety policies increases in educational laboratories compared to other laboratories as students often lack sufficient knowledge about the hazards possessed in the laboratory. Some education laboratories may have established safety rules but are not followed. According to a study done by Biehle and his colleagues (2007), the preponderance of education laboratories does not follow safety standards in the Kansas City region in USA.^13^

The same conclusion is evident or can be derived from several other studies around the world.^14^,^15^ Part of the solution for the educational laboratories’ safety problem is to promote a hazard-awareness culture and implement practical control measures.^16^ This should be achieved through proper risk management and applicable safety rules for the types of hazards possessed in the laboratory. The same concept was observed by Zaveri et al (2012); eliminating work-related hazards in laboratories necessitates a full awareness of the hazards and practical control measures to be implemented.^16^ As a result, there is a necessity to have proper risk management and applicable safety rules for the types of hazards possessed in the laboratory.

Qatar University (QU) is the national university in Qatar, and students of the BMS program are the future laboratory technologist in Qatar. Furthermore, BMS Department is preparing well-trained students to work in routine clinical and research laboratories in Qatar, who will be responsible for operating clinical laboratories at hospitals, primary care centers, and biomedical research laboratories at the national level after graduation. After the crisis of pandemic COVID-19, it becomes an urgent issue to address the risk and safety in biomedical laboratories at all levels to avoid the spread of such severe biological hazards and its consequences.

The main objective of this study was to evaluate the safety of BMS educational laboratories. To approach this objective, we identified the potential hazards and determined the actions or control measures required to eliminate or reduce any risks to the BMS students, teaching assistants, laboratory technicians, faculties, and other related workers, following an RM process.

## Materials and Methods

### Study Design

A prospective and retrospective cross-sectional study was conducted from January to March 2020 at the Biomedical Laboratory Science Department (BMS)- College of Health Sciences (CHS) at Qatar University (QU). Two education laboratories were selected and included, namely the Medical Microbiology (BIOM 322) and Hematology & Hemostasis (BIOM 451). These laboratories were chosen as they were the most active laboratory exposed to several types of hazards. These hazards include, for example, biological samples such as body fluids (serum and blood), microbial strains, chemical reagents, and various procedures and techniques used in these laboratories, which made them an ideal selection for the present study as a good model of risk assessment. The study was ethically approved by the BMS department-College of CHS-QU. Various tools were used to assess risks, such as brainstorming, inspection, risk matrix, and FMEA.

### Risk Assessment Tools

Oral interviews were held with the persons in charge of each of the selected laboratories. The interview aimed to explain the study’s objectives, collect the required documents, and ask well-prepared questions about the study. The required documents included laboratory manual of each course, laboratory safety manual, incident and violation forms, safety data sheet (SDS), chemical inventory, equipment inventory, previous inspection reports, equipment maintenance plan, and equipment maintenance record.

A table was prepared to include the name of collected and missing documents. The questions aimed to address the following topics; the sources of the samples, the types of risks present in the laboratory, work instruction sheet, vaccinations, student training, staff training, equipment maintenance plan, equipment maintenance record, the average number of people working in the laboratory daily (including students), emergency protocols, in campus health and safety staff contacts, previous accidents reports, health and safety inspection reports, prospective equipment, name of experiments, and name of microorganism. The safety data sheet (SDS) and the laboratory manual for each of the laboratories were compared, and the purpose was to find if the SDS folders were up to date or not. A checklist was prepared according to the SDSs. A separate checklist was used for each laboratory during the inspection. Data collection sheets (hazard identification sheet and hazard evaluation sheet) were prepared (see supplementary materials).

### Types of Hazards and Definitions

Physical hazards are present due to some laboratory operations that threaten employees due to the materials or equipment used.^17^ The risks include the following: compressed gases, high-pressure reactions, heat, stress, and noise. Also, workers face general hazards related to the workplace, which result from their activities inside the laboratory, such as falls and slips, wounds, and health problems caused by frequent routine movement.

Ergonomic hazards refer to physical conditions that may pose a risk of injury to the musculoskeletal system such as repetitive motion (eg, pipetting), prolonged awkward postures (eg, while using a microscope), insufficient lighting, lifting, pulling, pushing, and gripping equipment, and exposure to extreme temperatures.^18^

Chemical hazards are risks property or personnel face in scientific laboratories during experiments or throughout the transportation, handling, and storage of chemicals.^19^ These include the risk of a chemical spill, fire hazard from flammable chemicals, the risk of chemical explosions, hazardous chemical waste dumped in containers and sanitation facilities, the risk of a fall, leak, and blast of a compressed gas cylinder; and the risk of mixing incompatible chemicals during transport, use, storage or disposal.^20^

Biological hazards: their seriousness is a concern in laboratories that deal with microorganisms or contaminated materials.^21^ These risks are usually found in clinical and infectious disease research laboratories but may be found in other laboratories.^22^ The assessment of the severity of biological materials requires considering several factors, including the organism treated and the activities carried out on this organism.

Radiation hazards are concerned through working with or around radioactive materials or exposure to very high radiation levels such as ultraviolet (UV) radiation and x-rays.^23^

Electrical Hazard is a serious laboratory hazard that exposes workers to burns, electrocution, shock, fire, or explosion. For example, poor wiring and defective electric wires, wet hands, and outlets close to water.^24^

#### The Hazard Identification and Evaluation

The hazard identification sheet contains a description and classification of hazards, while the hazard evaluation sheet includes an estimation of likelihood, severity, and calculation of risk priority number (RPN). The likelihood and severity have five levels, as shown in Table 1. The RPN is calculated by multiplying the likelihoods of occurrence and the severity scores. Depending on the multiplication result, the hazard is classified into one of the four risk levels: high, warning, medium, and low, as shown in Table 1.

**Table 1:** The Risk Evaluation

Likelihood	Level	Occurrence Criteria
Frequent	5	Likely to occur many times per year
Moderate	4	Likely to occur once a year
Occasional	3	Might occur once in 3 years
Remote	2	Might occur once in 5 year
Unlikely	1	Might occur once in 10 years
Severity	Level	Occurrence criteria
Critical	5	Fatal/permanent injury; Poison/Infection with unknown cure; Spill outside campus; > $10 million damage; > 1-year downtime
Very serious	4	30 days medical certificate (MC)/hospitalization; Infection with known cure; Spill outside building; > $1 million damage; > 3-month downtime
Serious	3	10 days medical certificate (MC)/hospitalization; Injury with 1-month recovery; Spill outside Laboratory/room; $100,000 damage; > 1-month downtime
Marginal	2	3 days medical certificate (MC); Very mild exposure; Spill outside workplace; > $10,000 damage; > 5 days downtime
Negligible	1	First aid treatment only; mild/no exposure; Spill within workplace; < $5000 damage; No significant downtime
Score	Risk level	Action
16 ~ 25	High	Operation not permissible Stop operation and review control
12 ~ 15	Warning	High priority remedial action Implement additional controls immediately
8 ~ 10	Medium	Remedial action at appropriate time Proceed with care. Additional control is advised.
1 ~ 6	Low	Residual Risk/Risk acceptable No imminent dangers. Frequent review in the change of procedure, material, or environment

**Notes:** Adopted from: Tun, T. (2017). Biomedical Laboratory: Its Safety and Risk Management.

**Abbreviation:** MC, medical certificate.

The control measures were divided into two groups; these are adopted control measures and recommended control measures. The adopted control measures refer to the control measures that already exist in the laboratory. In contrast, the recommended control measures are not present, but they are required according to the assessment.

### Statistical Analysis

The data obtained from the data collection sheets (hazard identification sheet and hazard evaluation sheet) was coded and entered the computer for analysis using Microsoft Office for Mac (version 16.35). The present study’s data are descriptive of the risks, adopted measures, and control measures. A Chi-square test was used to detect any significant difference between the categorical variables of the two laboratories. The Two-tailed p-value is significant at≤ 0.05. SPSS program for Windows (version 23 statistical software; Texas instruments, IL, USA) was used for analysis, and the GraphPad Prism program was used to draw the figures (version 8, for Windows, Graph Pad Software, La Jolla California USA).

## Results

### Distribution of Hazards Types at the Microbiology and the Hematology Laboratories

The types and the frequency of the hazards were analyzed for each laboratory. In the microbiology laboratory, the ergonomic, physical, and chemical hazards accounted for 25% of all the laboratory hazards. Biological hazards constituted around 18% of hazards, followed by electrical hazards with 6.3%, while in both laboratories, there was no radiation hazard. Hematology laboratory showed similar findings; however, fewer physical hazards which equal biological hazards constituting 15.4% of the hazards (Table 2). There was no significant difference between microbiology and hematology laboratories regarding the type of hazard (*P*= 0.388).

**Table 2:** The Frequency Distribution and Percentage of Different Types of Hazards in Hematology and Microbiology Laboratories

Type of Hazard	Hematology Laboratory		Microbiology Laboratory	
	Count	Percent (%)	Count	Percent (%)
Chemical	4	30.8	4	25
Ergonomic	4	30.8	4	25
Biohazard	2	15.4	3	18.75
Physical	2	15.4	4	25
Electrical	1	7.6	1	6.25
Radiation	0	0	0	0
Total	13	100	16	100

**Note:** Data are presented as count and percent.

### The Risk Assessment and the Control Measures Adopted at the Microbiology and the Hematology Laboratories

**Figure 1:**
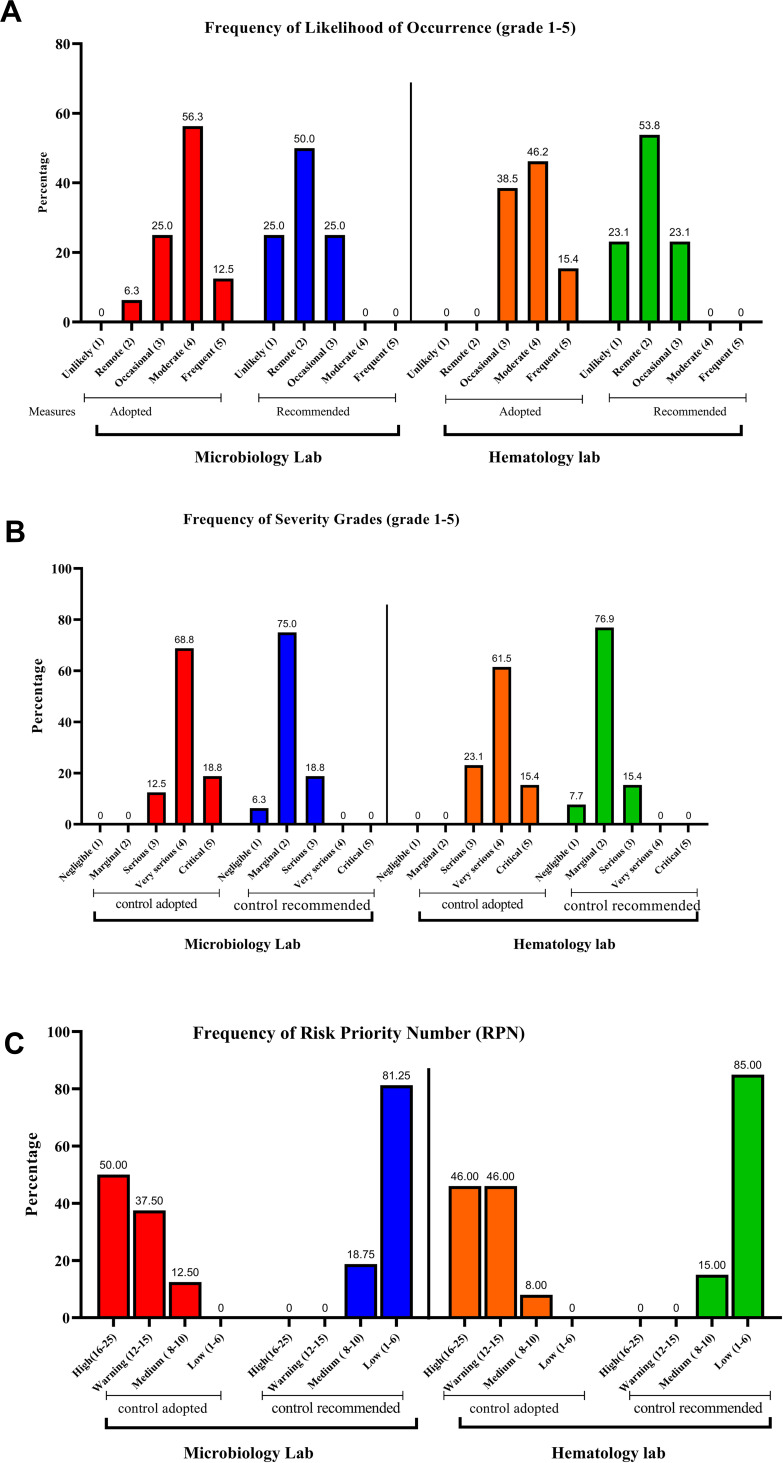
Bars represent the risk assessment for adopted and recommended control measures at the Microbiology (M) and Haematology (H) labs. (**A**) Bars represent the percentage of different grades of the likelihood. (**B**) Bars represent the percentage of different grades of severity. (**C**) Bars represent the percentage of different grades of Risk Priority Number (RPN).

Following the identification of the different types of hazards and their frequency in each laboratory, the risks were assessed based on the likelihood of occurrence, severity, and risk priority number based on the control measures adopted in each laboratory (Figure 1A–C).

#### The Assessment of Likelihood

Following the adopted control measures, the highest percentage of hazards in both laboratories had a moderate (4) likelihood, followed by occasional (3) and frequent (5) likelihood, respectively. None of the hazards in both laboratories had a likelihood of unlikely (1), and none had remote (2) likelihood in the hematology laboratory. Overall, there was a significant difference between microbiology and hematology laboratories in terms of the likelihood of occurrence (*P*= 0.0143) (Figure 1A).

#### The Assessment of Severity

Similar to the results of the likelihood of occurrence of hazards, all-hazards were distributed in the higher categories of severity. In the microbiology laboratory, most of the hazards were very serious (4), followed by critical (5) then serious (3). In the hematology laboratory, serious (3) severity hazards were more than those with critical (5) severity. There was no significant difference between microbiology and hematology laboratories regarding hazard severity (P= 0.164) (Figure 1B).

#### The Assessment of Risk Priority Number (RPN)

The RPN is the result of combining the likelihood and the severity of a hazard. Since the hazards identified in both laboratories had mostly high likelihoods and severities, the RPN of the identified hazards was naturally high. In the microbiology laboratory, 50% of the hazards had a high (16–25) RPN. More than 90% of the hazards had a high (16–25) or warning (12–15) RPN in the hematology laboratory. There was no significant difference between microbiology and hematology laboratories in terms of risk priority number (RPN) (P= 0.421) (Figure 1C).

#### The Risk Matrix for Adopted Control Measures and Risk Distribution

Table 3 displays The Risk Matrix (5x5) for adopted control measures and risk distribution at the microbiology and hematology laboratories. The categorical data of Microbiology laboratory (M) demonstrated that; one hazard (n=1, 6.25%) has a likelihood of moderate (4) and severity of critical (5). One hazard (n=1, 6.25%) has a likelihood of remote (2) and severity of very serious (4). Two hazards (n=2, 12.5%) has a likelihood of occasional (3) and severity of critical (5). Two hazards (n=2, 12.5%) has a likelihood of occasional (3) and severity of very serious (4). Two hazards (n=2, 12.5%) has a likelihood of moderate (4) and severity of serious (3). Two hazards (n=2, 12.5%) has a likelihood of frequent (5) and severity of very serious (4). Six hazards (n=6, 37.5%) has a likelihood of moderate (4) and severity of very serious (4).

**Table 3:** The Risk Matrix (5x5) for Adopted Control Measures and Risk Distribution at the Microbiology and Hematology Laboratories

Likelihood	Severity									
	Critical (5)		Very Serious (4)		Serious (3)		Marginal (2)		Negligible (1)	
	M	H	M	H	M	H	M	H	M	H
Frequent (5)			2							
Moderate (4)	1	2	6		2					
Occasional (3)	2		2							
Remote (2)			1							
Unlikely (1)										

**Notes:** Data are presented as numbers.

**Abbreviations:** M, microbiology laboratory; H, hematology laboratory.

Furthermore, Table 3 displays the categorical data of the Hematology laboratory (H), two hazards (n=2, 15.4%) had a likelihood of occasional (3) and severity of critical (5). Two hazards (n=2, 15.4%) had a likelihood of frequent (5) and severity of very serious (4). Two hazards (n=2, 15.4%) had a likelihood of moderate (4) and severity of serious (3). Three hazards (n=3, 23.1%) had a likelihood of occasional (3) and severity of very serious (4). Three hazards (n=3, 23.1%) had a likelihood of moderate (4) and severity of very serious (4). One hazard (n=1, 7.6%) had a likelihood of occasional (3) and severity of serious (3).

### The Risk Assessment and the Control Measures Recommended at the Microbiology and the Hematology Laboratories

#### The Assessment of Likelihood

The likelihood of identified hazard occurrence following the recommended control measures was remote (2) in about 50% of all hazards in both laboratories, and the remaining were divided equally between unlikely (1) and occasional (3) likelihood (Figure 1A).

There was no significant difference between microbiology and hematology laboratories regarding the likelihood of occurrence (*P*=0.851).

#### The Assessment of Severity

As for the severity, three-quarters of the hazards identified had a marginal (2) severity in both laboratories. None were very serious (4) or critical (5) (Figure 1B).

There was no significant difference between microbiology and hematology laboratories in terms of hazard severity (*P*= 0.676)

#### The Assessment of Risk Priority Number (RPN)

Most of all identified hazards following the recommended control measures had a low (1–6) RPN in both laboratories. The highest severity in both labs was medium (8–10), constituting 18.75% and 15% of hazards in microbiology and hematology labs. There was no significant difference between microbiology and hematology laboratories in terms of risk priority number (RPN) (P= 0.453) (Figure 1C).

#### The Risk Matrix for Recommended Control Measures and Risk Distribution (Table 4)

In the microbiology laboratory, one hazard (n=1, 6.25%) had the likelihood of occasional (3) and severity of serious (3), as shown in Table 4. One hazard (n=1,6.25%) had the probability of unlikely (1) and severity of critical (5). One hazard (n=1,6.25%) had the likelihood of remote (2) and severity of serious (3). One hazard (n=1,6.25%) had the possibility of unlikely (1) and severity of serious (3). One hazard (n=1,6.25%) had the likelihood of remote (2) and severity of negligible (1). Three hazards (n=3,18.75%) had the likelihood of occasional (3) and severity of marginal (2). Six hazards (n=6,37.5%) had the likelihood of remote (2) and the severity of marginal (2). Two hazards (n=2,12.5%) had the likelihood of unlikely (1) and severity of marginal (2).

**Table 4:** The Risk Matrix (5x5) for Recommended Control Measures and Risk Distribution at the Microbiology (M) and Hematology (H) Laboratories

Likelihood	Severity									
	Critical (5)		Very Serious (4)		Serious (3)		Marginal (2)		Negligible (1)	
	M	H	M	H	M	H	M	H	M	H
Frequent (5)										
Moderate (4)										
Occasional (3)					1	1	3	2		
Remote (2)					1	1	6	5	1	
Unlikely (1)	1				1		2	3		

**Note:** Data are presented as numbers.

**Abbreviations:** M, microbiology laboratory; H, hematology laboratory.

In the hematology laboratory, two hazards (n=2,15.4%) had a likelihood of occasional (3) and severity of marginal (2). One hazard (n=1,7.6%) had a probability of occasional (3) and severity of serious (3). One hazard (n=1,7.6%) had a likelihood of remote (2) and severity of serious (3). One hazard (n=1,7.6%) had a likelihood of remote (2) and severity of negligible (1). Three hazards (n=3,23.1%) had a likelihood of unlikely (1) and severity of marginal (2). Five hazards (n=5,38.7%) had a likelihood of remote (2) and severity of marginal (2), as shown in Table 4.

### Comparison Between Adopted and Recommended Control Measures at the Microbiology Laboratory

#### Assessment of Biosafety Level 2 (BSL2) Requirements

The number of statements in the biosafety level 2 (BSL2) checklist was 61. The microbiology laboratory approximately adhered only to half of the checklist requirement, with 52.5% (n=32) of the statement checked as yes and 47.5% (n=29) as no. The hematology laboratory’s percentage of adherence to BSL2 requirements is 68.9% (n= 42), while it failed in 31.1% (n=19) of them.

## Discussion

The educational biomedical laboratories have an array of unique hazards. These hazards include physical, ergonomic, chemical, biohazards, electrical hazards, and radiation facets.^21^ The importance of risk assessment in the educational biomedical laboratory increases worldwide and gaining more attention. These laboratories house different actors with different expertise, skills, knowledge, and education, such as students, teachers, scientists, administrative staff, and others. Generally, students lack a proper understanding of the hazards around them, how to deal with risks, lack of commitment, and adherence to security and safety rules, and many of them generate curiosity in dealing with all materials and equipment in the laboratory.

Furthermore, scientific experiments usually demand chemicals, fumes, heating sources, and other possibly hazardous variables. In addition, biomedical field studies utilize human and biological specimens from healthy subjects and patients with different diseases for training and educational purposes, which requires more attention. A previous study conducted by Peplow and Marris (2006) concluded that a relaxed approach toward safety is that academic laboratories make them more dangerous than industrial laboratories.^25^ The educational laboratories also consist of various actors such as students, teachers, scientists, administrative staff, and others with different skills, knowledge, and education.^26^ The outbreak of COVID-19 as a pandemic crisis worldwide raised the subject of safety in the biomedical laboratory as a major priority. Proper training of biomedical students should include technical laboratory skills and safety; this is critical as these students’ professional careers demand such knowledge. According to Meyer (2012), the emerging of new materials and technologies and the increasing complexity of laboratory activities necessitate the build of risk management as a part of routine laboratory processes.^26^ Biomedical students work after graduation in clinical laboratories at the hospitals, medical care centers, and biomedical research fields and should be aware of such risks. The presence of biosafety measures and awareness of how to handle risks is vital in the biomedical field. All potential risks must be identified, assessed, and controlled to have the proper safety procedures, referred to as the risk management process (RM).

The present study aimed to evaluate the risks encountered in two educational laboratories in the BMS dept to highlight the risks and safety adopted and use such results as feedback to improve BMS laboratories’ safety quality. The study was conducted to evaluate the safety and to identify potential hazards for Microbiology (M) and the Hematology (H) laboratories. The study concluded the actions or control measures required to eliminate or reduce any risks to the Biomedical Sciences (BMS) students, teaching assistants (TAs), laboratory Technicians, Faculties, and other related workers, following a Risk management (RM) process.

The results of the current study demonstrated three major findings as primary outcomes. First, chemical, and ergonomic hazards had the highest percentages of identified hazards in both Hematology and Microbiology laboratories, with a similar percentage of 31% and 25% of each hazard in each laboratory, respectively. Second, there was a gap between adopted and recommended control measures per each laboratory in terms of likelihood, severity, and the risk priority number (RPN), as shown in the hazard evaluation sheet. Third, the probability of occurrence for the adopted control measures showed a statically significant difference between microbiology and hematology laboratories (*p* = 0.0143).

The current study’s findings demonstrated that chemical and ergonomic hazards had the highest percentages in both laboratories for the distribution of hazard types, constituting about a quarter of the hazards present in each laboratory. The frequency of chemical and ergonomic hazards was 25% of each hazard type in the microbiology laboratory and 31% of each type in the hematology laboratory. The frequency of hazards types encountered in the hematology educational laboratories was chemical (31%), ergonomics (31%), biohazards (15%), physical hazards (15%), electrical hazards (8%), and radiation hazards (0%). While, in the microbiology laboratories was chemical (25%), ergonomics (25%), biohazards (18.8%), physical hazards (25%), electrical hazards (6.3%), and radiation hazards (0%). The physical, ergonomic, and chemical hazards had the highest percentages of the microbiology hazards, with an equal percentage of 25% of each hazard type.

Hazards to students and staff were nearly everywhere in both laboratories, which is not surprising given the diversity in experiments and the limited research conducted to improve students and staff safety in educational laboratories. The total number of hazards identified in both academic laboratories was 29 hazards. The percentages of different types of hazards in both BMS educational laboratories as follows: the chemical hazards were 27.6% (n=8), the ergonomic hazards were 27.6% (n=8), the biohazards were 20.7% (n=6), the physical hazards were 17.2% (n=5), the electrical hazards were 6.9% (n=2), and radiation hazards 0% (n=0).

A study conducted by Haile (2012) mentioned that laboratory workers are at risk of ergonomic injury while performing repetitive laboratory procedures such as pipetting, using cell counters, and working at microscopes.^27^ They observed that ergonomic injury is strongly associated with work-related musculoskeletal disorders (WMSDs). Also, they found that ergonomic hazard can be reduced by developing a comfortable working environment and applying ergonomic principles.

The high percentage of ergonomic hazards in both BMS educational laboratories could be due to improper adjusted working benches and chairs, poor posture, and repetitive movements. Such an issue is subjected to further studies to reduce ergonomic hazards and provide recommendations based on the administration’s feedback.

The percentage of biohazards identified is 18.75%, and electrical hazards are 6.25% and, radiation hazards are 0% in the microbiology laboratory. A previously published study by Thafer (2013) reported that biological and chemical hazards had the highest percentages of hazards, with 75% to biological hazards and 70% to chemical hazards. The difference in the results can be due to the academic year’s difference, type of experiments, and laboratory type, whether research, medical or educational laboratories. In the study conducted by Thafer, 164 medical laboratories were included from over 12 governmental hospitals. They used a self-administered questionnaire to collect data.^28^

Moreover, the comparison between adopted and recommended control measures showed a decrease in the severity, the likelihood of occurrence, and risk priority number (RPN) of the total 29 previously identified hazards. A significant difference between adopted and recommended control measures has been revealed in both laboratories in the present study. For example, hazard number six (R6) in a microbiology laboratory refers to exposure to BSL-2 biological agents during technical laboratory work such as reading culture plates, removing caps or swabs, subculturing, streaking plates. The likelihood has decreased from moderate (4) to remote (2) for spilling and splashing of hazardous chemicals by providing instructions on how to use the spill kits and wearing appropriate personal protective equipment (PPE). The severity has decreased from critical (5) to marginal (2)-for exposure to BSL-2 biological agents during reading culture plates, subculturing, and removing cap by working under biosafety cabinet (BSC class 2). This could be achieved by paying attention to immunocompromised persons, feeding mothers, and pregnant students and facility members. Also, implementing microbial control procedures such as providing a separate sink designated to hand washing only. The risk priority number (RPN) has decreased from high- RPN (20) to low- RPN (4)- for storage of flammable chemicals by ensuring suitable storage arrangement such as prohibiting overloading stored chemicals and using flammable chemicals cabinets.

Chemical management is an essential step to ensure a safe working environment throughout an educational laboratory. According to the audit investigation of both laboratories, there was a mismanagement of the chemicals inside. For example, flammable chemicals like Gram stain reagents were stored in large quantities in a wooden cabinet. According to Meyer (2012), storage of chemicals is considered an essential control measures to avoid unwanted results. They also mentioned that proper chemical management consists of three steps: ordering and authorization, inventory and storage, and finally, waste management.^26^

A study conducted by Gurses suggested that a weekly stock chemical should be kept inside the lab, and further storage should be managed elsewhere.^29^

The present study displays that microbiology and hematology laboratories are small, crowded, and suffering from inadequate physical space and delays in completing biohazard and sharp waste bins disposal, which impose a significantly high risk for accidents. The number of students and staff working in both microbiology and hematology laboratories in each session is 15 to 20 and 2 to 3, respectively. The two microbiology and hematology laboratories have the same space (9 x 5.5 m).

A study found that teaching and research laboratories can be too crowded, and such overcrowding increases spills’ risk. Also, the study mentioned that those labs were complaining about improper waste disposal such as dumping open chemical bottles in the domestic waste bin.^26^

An observational study using contextual inquiry and on-site photographing found poor work design creates an unnecessary increase in workload to workers and decreases situation awareness.^29^,^30^ The study also showed that insufficient horizontal space leads to improper supplies and equipment storage and makes reaching the necessary items hard. It also increases the chance of objects falling on the floor and bumping something.^29^,^30^ A study conducted by Gurses (2012) found that physical environment hazards such as layout problems, cluttered workspace, and poor location of equipment and supplies account for 16% (n=5) of the total number of identified hazards in the cardiovascular operating room (CVOR) (n= 31).^30^

There is no doubt that the presence of personal protective equipment (PPE) in the laboratory is very important, and no laboratory, whether educational or clinical, is devoid of them.^31^ However, the most important concept is the presence of knowledge of using and disposing of used PPE. According to a previous study conducted at an academic pediatric ambulatory clinic, the existence of knowledge of proper disposal of PPE among health care workers (HCWs) is not less important compared to wearing PPE in the laboratory, and this is because contaminated PPE can threat on other HCWs and a large scale public health and the surrounding environment. This study also concluded that there was a need for integrating Human Factors and Ergonomics (HFE) techniques and tools in the health care systems as a proactive method to decrease the risk of hazard exposure.

For example, hazard number five (R5) in the hematology laboratory, using real blood samples obtained from Hamad Hospital. The likelihood has decreased from occasional (3) to remote (2). The severity has decreased from serious (3) to marginal (2) using real blood samples obtained from Hamad hospital by maintaining regular test containment arrangement, working under a biosafety cabinet (BSC class 2). Furthermore, paying attention to unusual risks to immunocompromised persons, feeding mothers, and pregnant students and facility members was accomplished. The risk priority number (RPN) has been reduced from high-RPN (16) to low-RPN (2) for fire hazards caused by flammable chemicals such as Wright stain. This was accomplished using a fume hood when handling volatile explosive, chemical, and conforming and acknowledging control measures listed in the standard operating procedure (SOP) for the used chemicals. In support of this current finding, a recent study conducted by Thafer (2013) showed that the control of hazards reduced the occurrence of occupational diseases and accidents.^28^ Another study by Ajaz et al (2008) demonstrated similar findings to the current data that mounting safety-engineered strategies lead to a major decrease in laboratory injuries.^32^ According to Stein et al (2010), the necessary control measures such as continuous education, implementing standard precautions, immunization against hepatitis B, and the improvement of prevention guidelines for blood-borne infections are essential to be executed.^33^ These results match Zafar et al (2009), where a significant decrease in needlestick injuries ascribed to continuous emphasis on increasing awareness through consistent educational conferences.^34^

A total of eight (n=8) documents, including laboratory manual, standard operating procedure (SOP), laboratory safety manual, incident and violation form, chemicals Material Safety Data Sheet (MSDS), infectious substances MSDS, carbon dioxide MSDS, List of bacteria, and list of experiments, were received from the in-charge staff of microbiology laboratory. A total of four (n=4) documents, including laboratory manual, SOP, laboratory safety manual, and chemical MSDS, were received from the in-charge staff of the hematology laboratory. These documents are available to all students and staff at all levels in the laboratory. Most of these documents indicated the importance of being aware of various types of hazards and risks; however, none of the papers showed the relationship between risks and hazards. Some of the received documents emphasized that all students and laboratory workers should be proactive and list the potential hazards, which may have influenced them and the surrounding environment.

Proactive risk identification methods or PHA aim to reduce any accidents happening in the future by using various forecasting methods such as brainstorming and FMEA.^11^ Implementing proactive hazard identification in any laboratory is extremely important to identify safety concerns, hazards, and risks before they occur and to redesign the process in a way that can improve students’ and staff safety. Also, PHA opens the imagination of new risks, especially since several incidents that occur in the laboratory are sporadic and unpredictable in the educational laboratories. However, the collected documents showed little evidence of the consuming of proactive hazard identification methods such as audit, inspection, and survey, and no evidence of using “Failure Mode and Effect Analysis (FMEA),” “Hazard Operability (HAZOP,”),” system mapping approaches (SMAs) and “Structured What-if Technique (SWIFT)”.^35^,^36^ The very slow and sporadic adoption of PHA approaches in both laboratories could be due to lack of expertise in healthcare, lack of training, time-intensive, and the absence of office for ergonomic safety.^37^ In support of this current finding, a recent study conducted at a UK based hospital showed that the healthcare staff had few experienced using PHA to identify risks prospectively valuable challenges such as insufficient training, time, and staff constraints.^5^

In contrast to PHA, reactive risk identification methods attempt to reduce the tendency of similar accidents, which happened in the past being repeated in the future. These methods facilitate learning from past experiences, implementing roost cause analysis (RCA), and providing a benchmarkable dataset on past risks.^37^ The current data demonstrated that both laboratories were missing the following documents (n=7), previous incident report, incident report file, equipment inventory, equipment maintenance file, chemical inventory list, bacterial strain (for microbiology lab), and waste management SOP. Based on the incident investigation (retrospective method), both microbiology and hematology laboratories do not record incidents besides incident report sheets. The disinclined to report safety incidents could be due to the absence of previous accidents or the reliance there is no use in reporting the incident.^29^ A previous study stressed on retrospective analysis such as incident reports as the main tool for risk identification.^35^ The same research emphasized the significance of encouraging all staff and students to report all injuries regardless of how minor was without the fear of reprisal. Because having proper reporting and documentation of the incident, near miss, errors, and first aid injuries have several advantages such as engaging students in solving problems process and enabling institutes to proactively resolve before a costly or tragic incident takes place in the future.^35^

## Limitations

This study’s main limitation is the relatively small sample size, which could compromise the inferences’ statistical results. In addition, this study did not cover the role of risk-culture as an important control measure. It would have been insightful if the study’s laboratories were followed up after submitting the college administration’s findings to study and analyze the recommended control measure’s effect after their implementation.

## Conclusions

Biomedical laboratories are considered essential educational tools in the college of health sciences (CHS). These laboratories have several benefits for the student, such as permit students to see how science conceptions are implemented and cooperate more straightforwardly with the world. The present study is the first attempt to assess the risks encountered in educational laboratories (hematology and microbiology). The data obtained highlighted the risks and safety adopted. The results are used as feedback to improve BMS laboratories’ safety quality. This study showed that a quarter of hazards present in both laboratories are due to chemical and ergonomic hazards. Chemical and ergonomic hazards have the highest percentages of overall hazards in both laboratories, with an equal percentage of 25% of each hazard in the microbiology laboratory and with an equal percentage of 31% of each hazard in the hematology laboratory. The severity, likelihood of occurrence, and risk priority number (RPN) are higher in the microbiology laboratory than in the hematology laboratory. This study gave some recommendations about the currently adopted control measure.

## Recommendations

Moving on forward, based on data generated from the present study, the BMS department should focus its resources on implementing office for ergonomic safety to increase and spread awareness among students and staff. This particular step will ensure the use of proper chairs, benches, cabinets, pipettors, and microscope to eliminate musculoskeletal stress and protect against disorders related to joints and movements.

To reduce the severity of the identified hazards, the BMS department needs to ensure the laboratory safety equipment—including fume hood and biosafety cabinet class 2 (BSC 2) are available in the laboratory when handling any toxic or hazardous agent. This is the first step in securing a proper and safe laboratory environment and reducing the likelihood of hazards exposure.

The study revealed that implementing different policies is a crucial prerequisite for improving risk management within laboratories. Indeed, this will not only improve safety in education laboratories but will ensure that exposure to hazardous material and chemicals will be little or non-existent. On this basis, the BMS department should restrict entrance to only authorized personal such as laboratory technicians, students, and teaching faculty. They should also provide a work instruction sheet that outlines the recommended safe method of undertaking the laboratory test.

The documentation of laboratory files can be improved by organizing chemicals and biological agents in a safety data sheet (SDS) alphabetically using a common name to make it easier to find a particular one in a stressful situation. Moreover, BMS should keep a record of equipment maintenance to ensure the integrity of laboratory equipment.

The study has shown that immunocompromised persons, feeding mothers, and pregnant females face a higher risk of different hazardous material and chemicals than others due to a lack of policies and practices devoted to shielding them from hazards more prone to than others. Therefore, it is recommended to establish policies that target this group in particular to prevent or combat exposure to hazards.

The two laboratories can be improved by addressing several engineering controls such as designing multiple handwashing sinks, using proper furniture, eg, cabinet and chairs, using the appropriate color for the biohazard waste bin, and follow appropriate chemical storage practices.

## Prospective

In the future, additional studies are needed to be done to prove the findings of this study. For example, more research should be done to study the severity of each type of identified hazard. Also, the perception and knowledge of occupational hazards among students and persons in charge need to be explored. The sample size should be expanded to include other education laboratories in CHS or another institute. It might also be insightful to include and compare research laboratories where similar laboratory settings to those education laboratories are in place, but with different staff expertise and knowledge. Moreover, other methods such as Root Cause Failure Analysis (RCFA) could be compared to FMEA method or assessed in conjunction with it.

## Plain Summary

Safety in educational laboratories is one of the most crucial topics. All-hazards need to be identified, evaluated, and controlled whenever possible, following the risk management (RM) process. The awareness of risk and safety increased after the pandemic crisis of COVID 19. Educational laboratories for biomedical students are considered crucial educational means to teach and train future laboratory workers in clinical settings in the college of health sciences (CHS). This study is the first effort to evaluate the risks encountered in educational laboratories at Qatar University to highlight the risks and safety adopted and raise feedback to improve the quality of academic laboratories’ safety to protect students and staff, health care subjects, and faculties. This study demonstrated that chemical and ergonomic hazards represent a quarter of hazards encountered in both laboratories.

## Abbreviations

FMEA, failure mode and effects analysis; PPE, personal protective equipment; QU, Qatar University; BMS, Biomedical Sciences; COVID, Coronavirus Disease; CHS, College of Health Sciences; SDS, safety data sheet; RPN, risk priority number; RM, risk management; BSL2, Biosafety Level 2; Tas, teaching assistants; WMSDs, work-related musculoskeletal disorders; BSC, biosafety cabinet; SOP, standard operating procedure.

## Data Sharing Statement

The datasets used and/or analyzed during the current study are available from the corresponding author on reasonable request.
